# Full thermal ablation versus partial thermal ablation for secondary hyperparathyroidism: A meta-analysis.

**DOI:** 10.1097/MD.0000000000036422

**Published:** 2023-12-01

**Authors:** Lifeng Gong, Xiaowu Liu, Qichao Yang, Wei Jiang, Xiaoming Liu, Xianping Li, Wei Xu

**Affiliations:** a Department of Nephrology and Urology, Wujin Hospital Affiliated with Jiangsu University, Changzhou, China; b Department of Nephrology and Urology, The Wujin Clinical College of Xuzhou Medical University, Changzhou, China; c Department of Endocrinology, Wujin Hospital Affiliated with Jiangsu University, Changzhou, China; d Department of Endocrinology, The Wujin Clinical College of Xuzhou Medical University, Changzhou, China; e Department of Nephrology, People’s Hospital of Hainan Tibetan Autonomous Prefecture, Hainan Tibetan Autonomous Prefecture, Qinghai, China.

**Keywords:** full thermal ablation, meta-analysis, partial thermal ablation, secondary hyperparathyroidism

## Abstract

**Background:**

Regarding the thermal ablation treatment of refractory secondary hyperparathyroidism (SHPT), there is no consensus on the ablation range of the hyperplastic parathyroid gland. Therefore, this meta-analysis was conducted to evaluate the efficacy and complications between full and partial thermal ablation in patients with refractory SHPT.

**Methods:**

Databases including PubMed, EMbase, the Cochrane Library, CNKI (China National Knowledge Infrastructure), and Wanfang databases were searched from inception to July 1, 2023. Eligible studies comparing full thermal ablation and partial thermal ablation for SHPT were included. Data were analyzed using Review Manager Version 5.3.

**Results:**

Four studies were included in the meta-analysis. Three cohort studies and one randomized controlled trial involving 62 patients in the full thermal ablation group and 63 patients in the partial thermal ablation group were included. The serum parathyroid hormone (PTH), calcium, and phosphorus levels after full ablation were all lower than those after partial ablation (*P* < .05). There was no significant difference between the partial and full ablation groups concerning the incidence rate of severe hypocalcemia (*P* = .09). There was no significant difference between the partial and full ablation groups concerning symptom improvement, including bone joint pain, itching, and myasthenia (*P* < .05).

**Conclusion:**

Full ablation was superior to partial ablation in terms of reducing PTH, calcium and phosphorus levels. Full ablation might not significantly increase the incidence of severe hypocalcemia. Larger multicentre randomized controlled trials are necessary to confirm the conclusion.

## 1. Introduction

Secondary hyperparathyroidism (SHPT) is a common and serious complication of end-stage renal disease.^[1]^ It is characterized by elevated levels of parathyroid hormone (PTH) because of hypocalcemia, hyperphosphatemia, and vitamin D deficiency in end-stage renal disease.^[2]^ SHPT can cause vascular calcification, soft tissue calcification, bone pain and fracture.^[3,4]^ High PTH levels are associated with an increased risk of mortality.^[5,6]^ In the early stage, SHPT can be controlled with drug therapy. For refractory SHPT, parathyroidectomy (PTX) is recommended in practical guidelines.^[3]^ A network meta-analysis showed that total parathyroidectomy with autotransplantation (tPTX + AT) is recommended as the most effective surgical procedure for SHPT with minimal side effects.^[7]^ However, total parathyroidectomy (tPTX) is most likely to result in refractory hypocalcemia or hypoparathyroidism.^[7]^ Subtotal parathyroidectomy (sPTX) is most likely to cause recurrence of SHPT.^[7]^

Recently, ultrasound-guided thermal ablation, a minimally invasive treatment, has been used for the treatment of refractory SHPT.^[8–10]^ The thermal ablation treatment of SHPT involves ablating all parathyroid glands or ablating only part of parathyroid glands, but it is not possible to transplant part of parathyroid glands. It is not clear which is the better treatment between full thermal ablation and partial thermal ablation. Full ablation is equivalent to tPTX, and partial ablation is equivalent to sPTX. However, some studies showed that the result of full ablation versus partial ablation did not seem to be the same as that of tPTX versus sPTX. Therefore, this meta-analysis was conducted based on the published literature to evaluate the efficacy and complications of full thermal ablation and partial thermal ablation under ultrasound guidance in patients with refractory SHPT.

## 2. Materials and methods

### 2.1. Search strategy

Our meta-analysis was reported in line with the Preferred Reporting Items for Systematic Reviews and Meta-Analyses and Assessing the Methodological Quality of Systematic Reviews guidelines. Ethical approval was not necessary since our meta-analysis is a statistical analysis based on previous literature.

We searched PubMed, Embase, the Cochrane Library, CNKI (China National Knowledge Infrastructure), and the Wanfang database from inception to July 1, 2023. The combined text and MeSH terms included secondary hyperparathyroidism, full ablation, and partial ablation. In addition, the relevant references and cited papers were searched manually to identify additional studies meeting the inclusion criteria. There were no language restrictions.

### 2.2. Inclusion and exclusion criteria

The inclusion criteria were randomized controlled trials (RCTs), cohort or case–control studies; PTH levels (>600 pg/mL), persistent hypercalcaemia and hyperphosphatemia and poor response to medical therapy, parathyroid gland hyperplasia diagnosed by ultrasound or radionuclide imaging; comparison of outcomes between full thermal ablation and partial thermal ablation; and outcomes including at least one of the indicators: serum PTH, serum calcium, serum phosphorus, bone joint pain, itching, myasthenia and hypocalcemia. Visual Analogue Scales (VAS) were used to score symptoms such as bone joint pain, itching, and myasthenia. A score of 0 was asymptomatic, a score of 1 to 3 represented mild symptoms, a score of 4 to 6 represented tolerable symptoms but disturbed sleep, and a score of 7 to 10 represented intolerable symptoms that affected appetite and sleep.^[11]^

Exclusion criteria were case series, comments, reviews; patients with primary hyperparathyroidism or tertiary hyperparathyroidism; patients who had undergone surgical treatment; and a lack of relevant outcome data.

### 2.3. Data extraction and quality assessment

Data were extracted independently by 2 investigators (L.G. and Q.Y.) using standard data extraction forms. In the case of disagreement, a third investigator (W.X.) was consulted. We extracted characteristics including first author, year of publication, location, study design, sample size, mean age, sex, follow-up period, specific methods of ablation, and treatment outcomes. The Cochrane assessment tool was used to assess the quality of RCTs,^[12]^ whereas the Newcastle–Ottawa scale was used to assess nonrandomized studies.^[13]^

### 2.4. Statistical analysis

This meta-analysis was performed using Review Manager Version 5.3 (Cochrane Collaboration). We summarized treatment outcomes as odds ratios for categorical variables and weighted mean differences for continuous variables with 95% confidence intervals. *P* < .05 was considered statistically significant. We used the *I*^2^ statistic to assess heterogeneity among studies. We considered *I*^2^ > 50% and *P* < .10 to indicate significant heterogeneity. Meta-analysis with insignificant heterogeneity was performed using the fixed-effects model. For meta-analyses with significant heterogeneity, the random-effects model was used. Publication bias was assessed using subgroup analysis or sensitivity analysis.

## 3. Results

### 3.1. Study selection and characteristics

**Figure 1.**
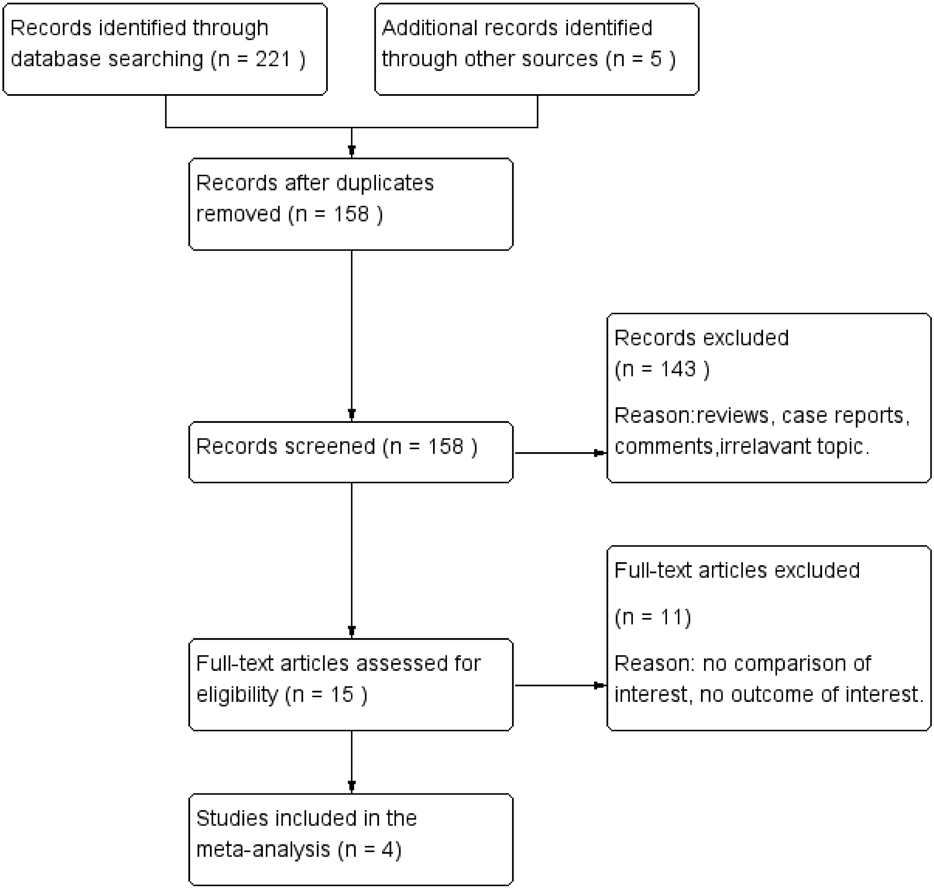
Flow diagram of the literature search.

A flow diagram of the selection process is shown in Figure 1. Finally, a total of 4 studies from China were included in this analysis.^[14–17]^ Of the 4 studies, 3 were cohort studies, and one was an RCT. As a whole, 62 patients were included in the full thermal ablation group, and 63 patients were included in the partial thermal ablation group. The follow-up period ranged from 1 month to 12 months. The risk of bias in the included RCTs was moderate. The cohort studies achieved scores of ≥ 6 points, which were of high quality. The baseline characteristics of these studies are listed in Table 1. The Cochrane assessment is listed in Table 2, and the Newcastle–Ottawa scale assessments are listed in Table 3.

**Table 1:** Characteristics of the included studies.

Study (year)	Country	Design	Follow-up period	Sample size	Mean age (yr)	Male (n, %)	PTH (pg/mL)	The method of partial ablation
Fuqiang Zeng 2021	China	RCT	12 months	Full ablation: 25 Partial ablation:25	54. 8 ± 8.7 55. 0 ± 8.5	15 (60.0) 16 (64.0)	1337.4 ± 483.3 1328.9 ± 411.6	If all 4 parathyroid glands could be detected, the smaller one with less blood supply should be retained. If less than 4 parathyroid glands were detected, half of the one with less blood supply should be retained.
Li Yu 2015	China	Cohort study	9 months	Full ablation:11 Partial ablation:15	52	16 (61.5)	1720.5 ± 750.7 1836.7 ± 661.0	The same as Fuqiang Zeng 2021
Chengzhong Peng 2014	China	Cohort study	1 months	Full ablation:9 Partial ablation:8	51.2 ± 8.8	2 (11.8)	1634.6 ± 352.9 1898.0 ± 691.5	3-5 parathyroid glands could be detected,2–3 were ablated and 1–2 were retained.
Jianchuan Yang 2018	China	Cohort study	6 months	Full ablation:17 Partial ablation:15	50.1 ± 11.7 48.8 ± 13.7	11 (64.0) 10 (66.7)	2040.7 ± l061.0 1909.0 ± 1009.4	Hyperplasia of parathyroid tissue showed less than 10% contrast agent filling.

**Table 2:** Quality assessment of randomized control trial.

Study	Random sequence generation	Allocation concealment	Blinding of participants and personnel	Incomplete outcome data	Selective reporting	Other bias
Fuqiang Zeng 2021	+	?	?	+	+	?

The randomized control trial was evaluated using the Cochrane assessment tool.

+, low risk of bias; ?, unclear risk of bias; -, high risk of bias.

**Table 3:** Quality assessment of cohort studies.

Studies	Selection	Comparability	Outcome	Score
Chengzhong Peng 2014	★★★	★	★★	6
Jianchuan Yang 2018	★★★★	★	★★	7
Li Yu 2015	★★★	★	★★	6

The Cohort studies were evaluated using the Newcastle-Ottawa scale, which are comprised of the study of selection (Representativeness of the exposed group, Representativeness of the non exposed group, Ascertainment of exposure, Demonstration that outcome of interest was not present at start of study), group comparability(Controls for the most important factor, Controls for any additional factor),outcome measures (Assessment of outcome, Was follow-up long enough for outcomes to occur, Adequacy of follow up of cohorts), a total of nine points. ★, 1 point.

### 3.2. Meta-analysis results

#### 3.2.1. PTH level.

**Figure 2.**
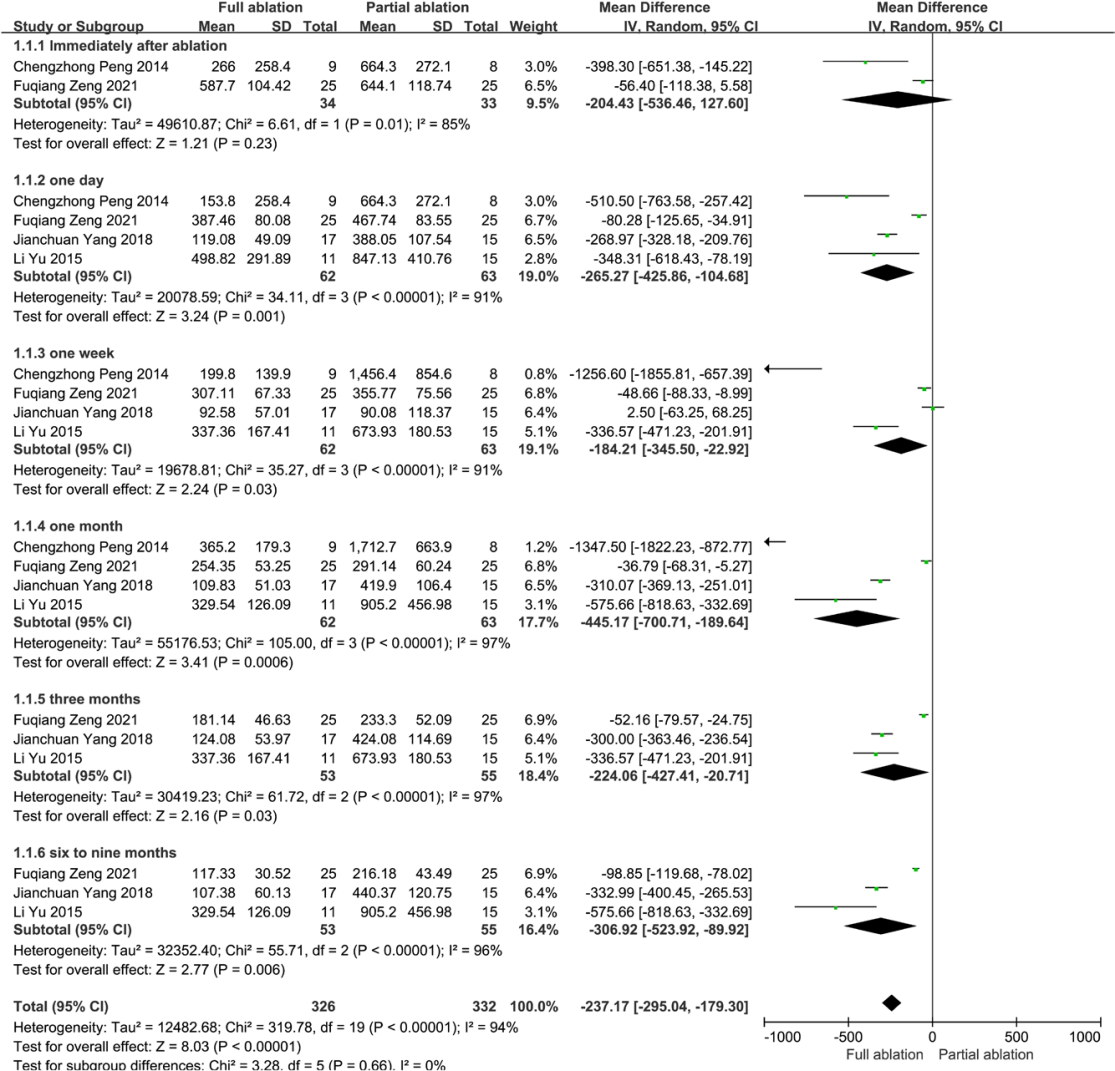
Forest plots comparing PTH levels between full ablation group and partial ablation group.

Data about PTH levels after ablation were reported in all articles.^[14–17]^ There was significant heterogeneity among the studies (*P* < .01, *I*^2^ = 94%), so the random-effects model was used for the meta-analysis. There was no significant difference between the partial ablation and full ablation groups concerning PTH levels immediately after ablation (*P* = .23). As a whole, PTH levels within 9 months after partial ablation were higher than those after full ablation, and the difference was statistically significant (*P* < .05) (Fig. 2).

#### 3.2.2. Calcium level.

**Figure 3.**
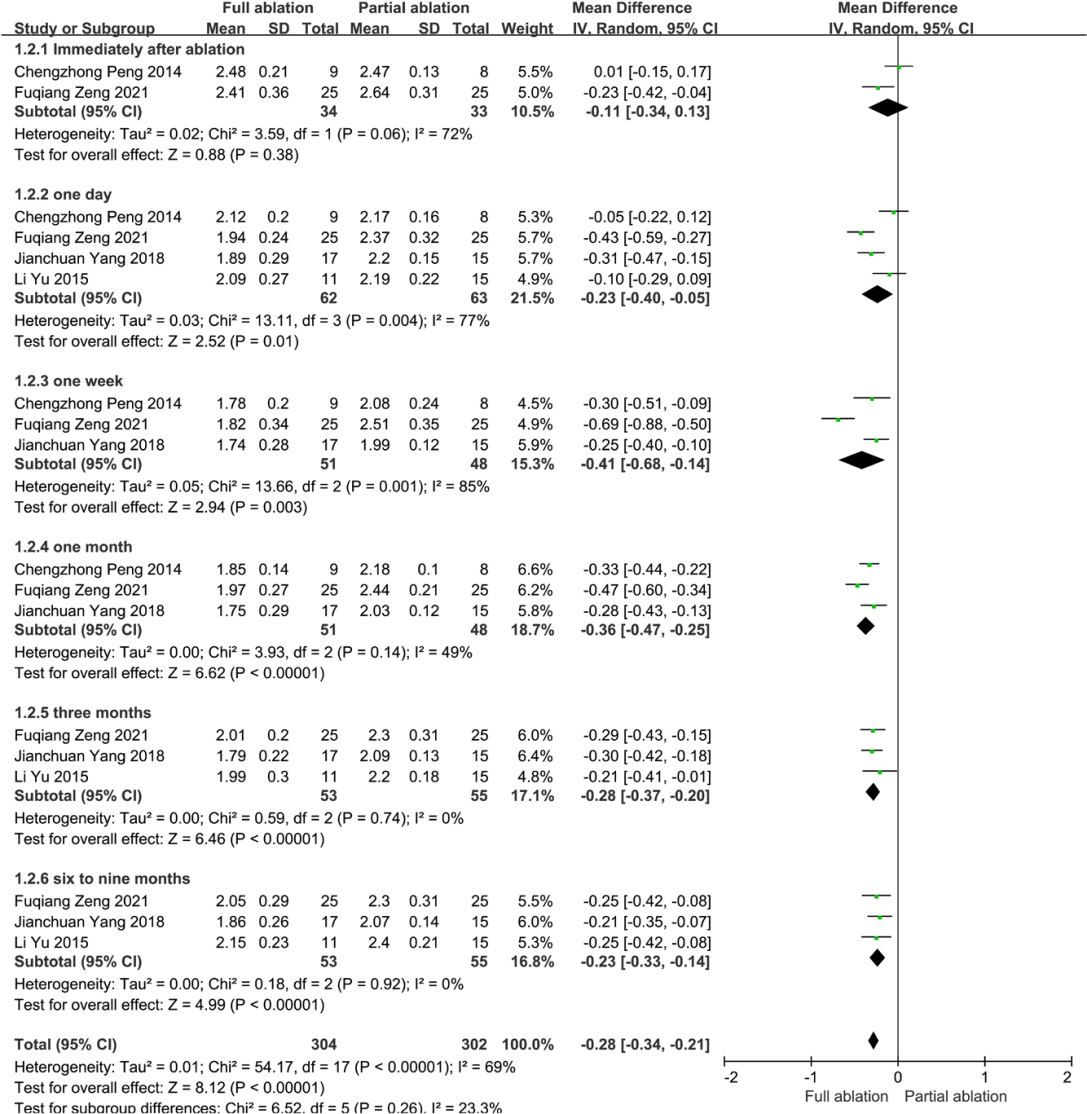
Forest plots comparing calcium levels between full ablation group and partial ablation group.

Data about calcium levels after ablation were reported in 4 articles.^[14–17]^ The total heterogeneity between these studies was significant (*P* < .01, *I*^2^ = 69%), so the random-effects model was used for the meta-analysis. There was no significant difference between the partial ablation and full ablation groups concerning calcium levels immediately after ablation (*P* = .38). As a whole, calcium levels within 9 months after full ablation were lower than those after partial ablation, and the difference was statistically significant (*P* < .05) (Fig. 3). However, the heterogeneity among the studies comparing calcium levels at 1 month, 3 months, and 6 to 9 months after ablation was not significant, so we used a fixed-effects model for the meta-analysis, which still showed that calcium levels at 1 to 9 months after full ablation were statistically lower than those at partial ablation.

#### 3.2.3. Phosphorus level.

**Figure 4.**
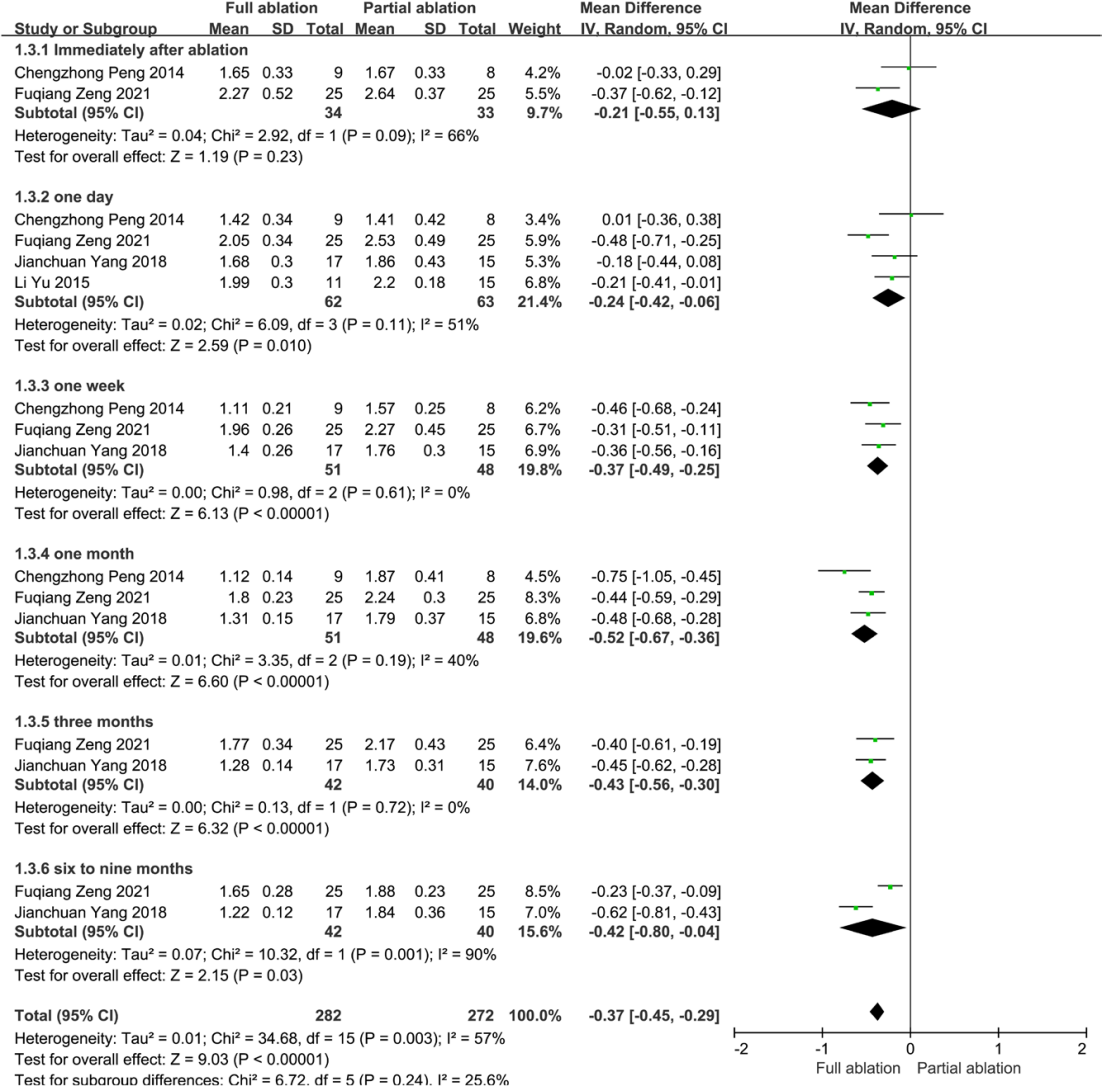
Forest plots comparing phosphorus levels between full ablation group and partial ablation group.

Data about phosphorus levels after ablation were reported in 4 articles.^[14–17]^ The total heterogeneity between these studies was significant (*P* < .01, *I*^2^ = 57%), so the random-effects model was used for the meta-analysis. There was no significant difference between the partial ablation and full ablation groups concerning phosphorus levels immediately after ablation (*P* = .23). As a whole, phosphorus levels within 9 months after full ablation were lower than those after partial ablation, and the difference was statistically significant (*P* < .05) (Fig. 4). However, the heterogeneity among the studies comparing phosphorus levels at 1 week, 1 month, and 3 months after ablation was not significant, so we used a fixed-effects model for the meta-analysis, which still showed that phosphorus levels at 1 week to 3 months after full ablation were statistically lower than those at partial ablation.

#### 3.2.4. Bone joint pain.

**Figure 5.**
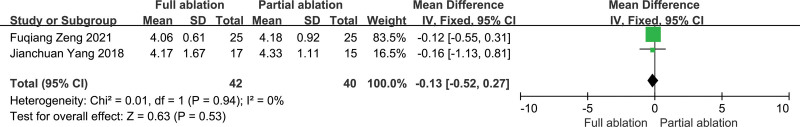
Forest plots comparing bone joint pain between full ablation group and partial ablation group.

Two studies reported data about the symptom scores of bone joint pain after ablation.^[15,17]^ The heterogeneity between these studies was not substantial (*P* = .94, *I*^2^ = 0%), so the fixed-effects model was used for the meta-analysis. The symptom scores of bone joint pain after full ablation were lower than those after partial ablation, but the difference was not statistically significant (*P* = .53) (Fig. 5).

#### 3.2.5. Itching.

**Figure 6.**
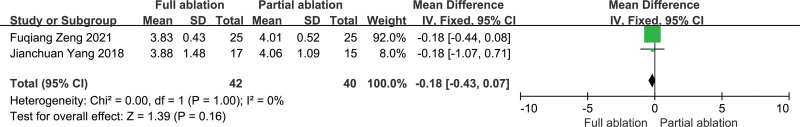
Forest plots comparing itching between full ablation group and partial ablation group.

Two studies reported data about the symptom scores of itching after ablation.^[15,17]^ The heterogeneity between these studies was not substantial (*P* = 1.00, *I*^2^ = 0%), so the fixed-effects model was used for the meta-analysis. The symptom scores of itching after full ablation were lower than those after partial ablation, but the difference was not statistically significant (*P* = .16) (Fig. 6).

#### 3.2.6. Myasthenia.

**Figure 7.**
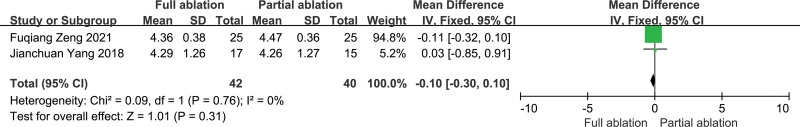
Forest plots comparing myasthenia between full ablation group and partial ablation group.

Two studies reported data about the symptom scores of myasthenia after ablation.^[15,17]^ The heterogeneity between these studies was not substantial (*P* = .76, *I*^2^ = 0%), so the fixed-effects model was used for the meta-analysis. There was no significant difference between the partial ablation and full ablation groups concerning the symptom scores of myasthenia after ablation (*P* = .31) (Fig. 7).

#### 3.2.7. Severe hypocalcaemia.

**Figure 8.**
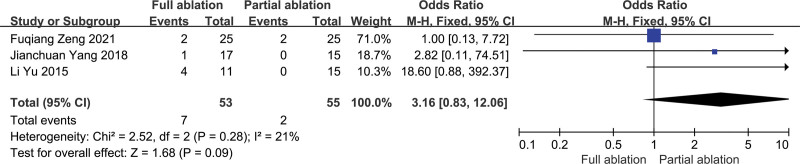
Forest plots comparing severe hypocalcemia between full ablation group and partial ablation group.

Three studies reported data about the incidence rate of severe hypocalcemia, 7/53 (13.2%) for the full ablation group and 2/55 (3.6%) for the partial ablation group.^[14,15,17]^ The heterogeneity between these studies was not substantial (*P* = .28, *I*^2^ = 44%), so the fixed-effects model was used for the meta-analysis. The incidence rate of severe hypocalcemia after full ablation was higher than that after partial ablation, but the difference was not statistically significant (*P* = .09) (Fig. 8).

### 3.3. Sensitivity analyses

A sensitivity analysis for the incidence rate of hypocalcemia and the symptom scores of bone joint pain, itching, and myasthenia after full and partial ablation treatments were used to judge the dependability of the result. We deleted one of all studies at a time, the heterogeneity was still not significant, and the results still showed no difference.

## 4. Discussion

Regarding the treatment of SHPT, tPTX is more likely to cause refractory hypocalcemia or hypoparathyroidism, and sPTX is more likely to cause recurrence.^[7]^ However, there is no consensus on the ablation range of the hyperplastic parathyroid gland. Our meta-analysis showed that full ablation was superior to partial ablation in terms of reducing PTH, calcium and phosphorus levels. Full ablation did not significantly increase the incidence of severe hypocalcemia, which was not the same as the result of tPTX versus sPTX in a previous meta-analysis. In addition, there was no significant difference between full and partial ablation concerning symptom improvement, including bone joint pain, itching, and myasthenia.

PTH levels after thermal ablation are an important indicator related to clinical efficacy. It was certain that full and partial ablation were both beneficial to decreasing PTH levels. During follow-up and at the end points, our meta-analysis showed that full ablation was superior to partial ablation in terms of reducing PTH levels. In the included research of Yu and Peng, PTH levels were still above 600 pg/mL after partial ablation, and partial ablation of the parathyroid was likely to have postoperative relapse. The reason may be that more parathyroid glands after partial ablation were preserved in their study of Yu and Peng than in the other included studies.

In terms of reducing calcium and phosphorus levels after thermal ablation, our meta-analysis showed that full ablation was superior to partial ablation. At the same time, our meta-analysis showed that full ablation did not significantly increase the incidence of severe hypocalcemia. However, in the included research of Yu, Yang and Peng, the average calcium levels were below 1.85 mmol/L, which occurred 1 week after full ablation. The reason for the low average calcium levels after full ablation was the sudden and large drop in PTH levels.^[18,19]^ The calcium level remained relatively high after partial ablation because the PTH value remained at a relatively high level after partial ablation. Almost all patients with hypocalcemia were relieved by calcium supplementation, and only one patient had persistent hypocalcemia in the full ablation group.

Overall, our meta-analysis showed that full ablation was superior to partial ablation. In terms of the traditional surgical procedures of SPTH, some experts did not recommend total parathyroidectomy (tPTX) because tPTX might result in refractory hypocalcemia, long-term low PTH levels and low turnover bone disease.^[20,21]^ In theory, full ablation of the parathyroid is equivalent to tPTX. However, in the included meta-analysis, refractory hypocalcemia was rarely reported in the treatment of full ablation. The reasons were as follows. First, some parathyroid tissues might not be completely ablated and may still have residue due to operator experience. Ablation is not performed under direct vision, so it may be less thorough than open surgical resection.^[9]^ Second, the parathyroid gland was not found by preoperative imaging, leading to missing ablation. Third, parathyroid ectopic glands or other tissues secrete PTH or similar bioactive substances, which can compensate for postoperative parathyroid function and partially maintain the stability of blood calcium. More studies are needed to confirm that full ablation did not significantly increase the incidence of severe hypocalcemia. In addition, the events of long-term low PTH levels and low transforming bone disease were not noticed as an outcome in the included research study, and we do not know their incidence.

There were some limitations in our meta-analysis. First, our meta-analysis showed that full ablation of the parathyroid reduced serum PTH and calcium more significantly. Full ablation did not significantly increase the incidence of severe hypocalcemia. However, there are no data about the events of long-term low PTH levels and low turnover bone disease after full ablation. Second, the definition of severe hypocalcemia is also unclear in the included research study of our meta-analysis. Third, the article by Li Yu did not describe whether the patient had ectopic parathyroid glands. At the same time, the exclusion criteria of patients in Li Yu’s study did not include ectopic parathyroid glands. Other studies excluded patients with ectopic parathyroid glands. So we added a sensitivity analysis. After deleting the study of Li Yu, we found that there was no difference in serum phosphorus levels between the full and partial ablation groups on the first day after ablation. Other outcomes concerning serum PTH, serum calcium and hypocalcemia did not change.

We propose further prospects. Appropriate preservation of hyperplastic parathyroid glands may also be a good choice for partial ablation treatment of SHPT based on the principle of safe medicine, since thermal ablation can be easily repeated and there are far more drugs available for SHPT than for hypocalcemia. In the included research of Peng, the patients with 1 or 2 glands retained had a small decrease in PTH and a rapid rebound. How many glands to retain and how to retain to control SHPT without recurrence and prevent hypocalcemia should be further discussed as a follow-up study.

## 5. Conclusions

Our meta-analysis revealed that full ablation reduced the serum PTH, calcium and phosphorus levels more significantly than partial ablation. However, full ablation did not significantly increase the incidence of severe hypocalcemia, which needs to be further verified. There was no significant difference between full and partial ablation concerning symptom improvement, including bone joint pain, itching, and myasthenia. To further confirm this conclusion, larger multicentre randomized controlled trials comparing full ablation and partial ablation for SHPT treatment are necessary.

## Acknowledgments

All authors gives permission to be named.

## Author contributions

**Conceptualization:** Lifeng Gong, Xiaowu Liu, Qichao Yang, Wei Xu.

**Data curation:** Xiaowu Liu, Wei Jiang, Xiaoming Liu, Xianping Li, Wei Xu.

**Funding acquisition:** Wei Xu.

**Investigation:** Lifeng Gong, Qichao Yang.

**Methodology:** Lifeng Gong, Xiaowu Liu, Qichao Yang, Wei Xu.

**Software:** Wei Xu.

**Supervision:** Lifeng Gong, Wei Xu.

**Writing – original draft:** Wei Jiang, Xiaoming Liu, Xianping Li, Wei Xu.

**Writing – review & editing:** Wei Jiang, Xiaoming Liu, Xianping Li, Wei Xu.
